# Development of attenuated live vaccine candidates against swine brucellosis in a non-zoonotic *B. suis* biovar 2 background

**DOI:** 10.1186/s13567-020-00815-8

**Published:** 2020-07-23

**Authors:** Beatriz Aragón-Aranda, María Jesús de Miguel, Leticia Lázaro-Antón, Miriam Salvador-Bescós, Amaia Zúñiga-Ripa, Ignacio Moriyón, Maite Iriarte, Pilar M. Muñoz, Raquel Conde-Álvarez

**Affiliations:** 1 Instituto de Salud Tropical (ISTUN), Instituto de Investigación Sanitaria de Navarra (IdiSNA) and Dpto. de Microbiología y Parasitología, Universidad de Navarra, c/Irunlarrea 1, 31008 Pamplona, Spain; 2 Unidad de Producción y Sanidad Animal, Centro de Investigación y Tecnología Agroalimentaria de Aragón (CITA), Avda. Montañana 930, 50059 Zaragoza, Spain; 3 Instituto Agroalimentario de Aragón-IA2 (CITA-Universidad de Zaragoza), Zaragoza, Spain

## Abstract

*Brucella* is a genus of gram-negative bacteria that cause brucellosis*. B. abortus* and *B. melitensis* infect domestic ruminants while *B. suis* (biovars 1–3) infect swine, and all these bacteria but *B. suis* biovar 2 are zoonotic. Live attenuated *B. abortus* S19 and *B. melitensis* Rev1 are effective vaccines in domestic ruminants, though both can infect humans. However, there is no swine brucellosis vaccine. Here, we investigated the potential use as vaccines of *B. suis* biovar 2 rough (R) lipopolysaccharide (LPS) mutants totally lacking O-chain (Bs2Δ*wbkF*) or only producing internal O-chain precursors (Bs2Δ*wzm*) and mutants with a smooth (S) LPS defective in the core lateral branch (Bs2Δ*wadB* and Bs2Δ*wadD*). We also investigated mutants in the pyruvate phosphate dikinase (Bs2Δ*ppdK*) and phosphoenolpyruvate carboxykinase (Bs2Δ*pckA*) genes encoding enzymes bridging phosphoenolpyruvate and the tricarboxylic acid cycle. When tested in the OIE mouse model at the recommended R or S vaccine doses (10^8^ and 10^5^ CFU, respectively), CFU/spleen of all LPS mutants were reduced with respect to the wild type and decreased faster for the R than for the S mutants. At those doses, protection against *B. suis* was similar for Bs2Δ*wbkF*, Bs2Δ*wzm,* Bs2Δ*wadB* and the Rev1 control (10^5^ CFU). As described before for *B. abortus*, *B. suis* biovar 2 carried a disabled *pckA* so that a double mutant Bs2Δ*ppdK*Δ*pckA* had the same metabolic phenotype as Bs2Δ*ppdK* and *ppdK* mutation was enough to generate attenuation. At 10^5^ CFU, Bs2Δ*ppdK* also conferred the same protection as Rev1. As compared to other *B. suis* vaccine candidates described before, the mutants described here simultaneously carry irreversible deletions easy to identify as vaccine markers, lack antibiotic-resistance markers and were obtained in a non-zoonotic background. Since R vaccines should not elicit antibodies to the S-LPS and *wzm* mutants carry immunogenic O-chain precursors and did not improve Bs2Δ*wbkF*, the latter seems a better R vaccine candidate than Bs2Δ*wzm*. However, taking into account that all R vaccines interfere in ELISA and other widely used assays, whether Bs2Δ*wbkF* is advantageous over Bs2Δ*wadB* or Bs2Δ*ppdK* requires experiments in the natural host.

## Introduction

Brucellosis is a worldwide extended zoonosis caused by gram-negative bacteria of the genus *Brucella*. This genus includes several nominal species among which *B. melitensis* preferentially infects small ruminants, *B. abortus* cattle and *B. suis* swine and semi-domestic and wild mammals [1]. These three species have been classically divided into biovars following phenotypic criteria [2], and out of the five biovars currently distinguished within *B. suis,* three infect domestic pigs. Biovars 1 and 3 are endemic in America and Asia, affect mainly domestic (and feral) pigs and wild boars and are very pathogenic for humans. *B. suis* biovar 2 (henceforth bv2) causes an enzootic infection in wild boars and also in hares in continental Europe. However, in contrast to other biovars infecting swine, bv2 shows a very reduced pathogenicity (if any) for humans. Indeed, the few infections reported only affect individuals with predisposing comorbidities that have been highly exposed [3, 4]. Although European Union countries are considered as free of porcine brucellosis, contacts between wild-life animals and domestic pigs occur in outdoor breeding systems and back yard herds, causing brucellosis outbreaks and subsequent long-term reproductive failures and economic losses [4]. In addition, *B. suis* bv2 can be introduced into intensive pig farms through infected replacements and/or semen [3].

In most cases, the complex and surreptitious dynamics of brucellosis makes the use of effective vaccines a requisite for its control and eradication in domestic animals [5]. But for a few instances where circumstances were highly favorable, the use of the *B. abortus* S19 (cattle live vaccine) and *B. melitensis* Rev1 (sheep and goat live vaccine) has been decisive wherever eradication has been achieved in domestic ruminants [6]. However, few studies have investigated brucellosis vaccines in swine, and none has been satisfactory. Both controlled experiments and field observations soon discarded *B. abortus* S19 as a useful brucellosis vaccine in pigs [7, 8] and, despite initial claims on full protection by the rough (R) (i.e. lacking the O-polysaccharide [O-chain] of the lipopolysaccharide [LPS]) *B. abortus* RB51 cattle vaccine [9], controlled experiments prove that this strain does not provide any protection to gilts [10]. Vaccination with a *B. abortus* attenuated strain (“Bang Viejo”) and the simultaneous injection of a crude heat extract of *B. suis*, plus a booster with the latter 1 month later, was reported to provide protection [11]. However, further experiments under controlled conditions found that this vaccination procedure (named INTA vaccine) does not protect pigs [11]. Attempts to develop a *B. suis* specific vaccine have been made using *B. suis* biovar 1 as background. In early studies, an apparently attenuated strain (Australian King 8) induced some protection at 6 months but none at 24 months after vaccination, which together with concerns about its reversion to full virulence and likely pathogenicity to humans, discarded its use as a vaccine [7]. *B. suis* strain 2 (also a biovar 1 derivative obtained by serial passage in vitro) has been claimed to be very useful in swine, and also in sheep, goats and cattle, and has been used in China since 1971. There is little experience with strain 2 outside of this country and, although it has been reported that its use led to brucellosis control in several areas of China [12], this claim is neither compatible with the present situation of the disease in the country [13] nor with the assessments made in European laboratories under controlled conditions in sheep [14, 15]. To the best of our knowledge, only a subunit vaccine has been tested in pigs. Edens and Foster [16] studied materials in the water phase (erroneously assumed to be rich in LPS) of a phenol/water extract of *B. suis* and reported protection. However, the data show protection inconsistent with the doses tested, which suggests that the bacteriological procedures (only a lymph node of the cervical region was examined for the challenge strain) were not optimized.

Although not tested in pigs, there have also been attempts to develop *B. suis* vaccines taking advantage of a better understanding of *Brucella* virulence factors and antigens. *B. abortus* and *B. melitensis* R mutants defective in the O-chain or in the O-chain plus core sugars of the LPS are attenuated and have been extensively investigated as a way to diminish the interference of ruminant vaccines in standard serological tests [17–20]. However, only Pgm (phosphoglucomutase involved in the production of the UDP-glucose involved in bridging the LPS core to the O-chain and in the synthesis of the periplasmic glucans [21]) and WboA (O-chain glycosyltransferase) have been mutated for developing a *B. suis* vaccine, either in a *B. suis* bv1 strain [21–23] or in *B. neotomae* [24]. Whereas both types of mutants yielded some protection in mice (see “Discussion”), both are made in brucellae that are infectious for humans and do not fully explore the possibilities offered by our current understanding of the genetics and structure of *Brucella* LPS. RB51 carries also a disrupted *wboA* [19] and it has been manipulated in attempts to bolster its performance either by overexpressing proteins thought to be immunogenic or involved in virulence [25] and/or by restoring the ability to induce anti-S-LPS antibodies [25, 26]. Yet, RB51 carries unknown genetic defects affecting LPS [19], which together with its poor performance in pigs [10] indicate that it is not an optimal background for developing a swine brucellosis vaccine. Moreover, RB51 (and hence other less attenuated R vaccines) is infectious for humans, and in this context it poses two additional problems: its resistance to rifampicin (used to treat human brucellosis) and undetectability of the infection in standard brucellosis serological tests [27].

**Figure 1:**
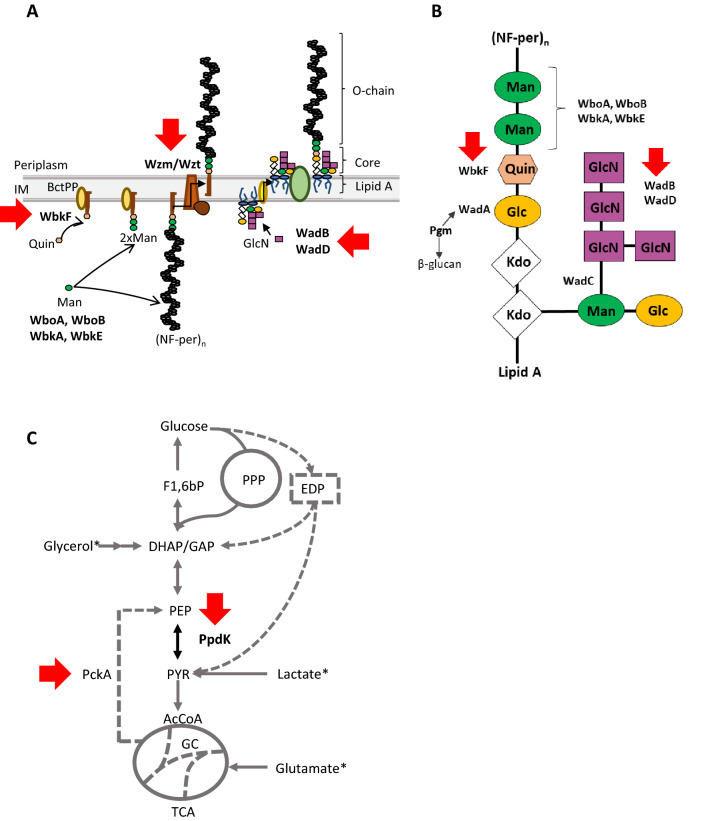
**Pathways and key enzymes targeted for the development of*B. suis*vaccines.** Schematic representation of *Brucella* (**A**) LPS biosynthesis steps that occur at both sides of the inner membrane (IM), **B** LPS core, and **C** central carbon metabolism. The proteins corresponding to the mutants investigated are indicated with arrows. WbkF, bactoprenol (BctPP, Bactoprenol-P-P) primase for O-chain polymerization; Wzm/Wzt, O-chain translocation ABC transport system; WadA, core glycosyltransferase that incorporates the terminal glucose (Glc) linking the core to the two O-chain mannoses (Man) of the proximal section of the N-formyl-perosamine (NF-per) polysaccharide; Pgm, phosphoglucomutase necessary for the synthesis of the UPD-glucose used by WadA; WadB, WadC and WadD, glycosyltransferases involved in the incorporation of glucosamine (GlcN) and Man to the core-lateral branch; Kdo, 3-deoxy-d-manno-octulosonic acid; Quin, quinovosamine. WboA, WboB, WbkA and WbkE, O-chain glycosyltransferases. F1,6bP, fructose-1,6-bisphosphate; DHAP, dihydroxyacetone-phosphate; GAP, glyceraldehyde-3-phosphate; PEP, phosphoenolpyruvate; PYR, pyruvate; AcCoA, acetyl-CoA; PckA, phosphoenolpyruvate carboxykinase; PpdK, pyruvate phosphate dikinase; EDP, Entner–Doudoroff pathway; PPP, pentose phosphate pathway; TCA, tricarboxylic acid cycle; GC, glyoxylate bypass. Asterisks mark substrates in the mGSM. The functionality of pathways represented as discontinuous lines varies depending on the *Brucella* species and biovar [17, 29–34, 56].

In this work, we investigated *B. suis* mutants that have three different types of LPS: carrying a complete LPS core but lacking the O-chain, carrying a complete LPS core and producing an internal O-chain, and mutants carrying an LPS with O-chain (i.e. a smooth [S] LPS) but defective in the core lateral branch (Figure 1A–B), three complementary strategies that produce attenuation and that respectively abrogate or not O-chain antibody production and enhance the immune response (Table 1) [17, 18, 28–31]. Moreover, no attempts to develop an attenuated *B. suis* vaccine have been made by exploiting our current understanding of *Brucella* metabolism [32], and here we also investigated *B. suis* bv2 vaccines lacking pyruvate phosphate dikinase (PpdK) and/or phosphoenolpyruvate carboxykinase (PckA) activities (Table 1 and Figure 1C) [33, 34]. We conducted our research in a *B. suis* bv2 strain because, since this biovar is not zoonotic, it is particularly appropriate for developing a vaccine posing no risks for humans.

**Table 1:** **Genes studied in the present work.**

Gene	Coding for	LPS phenotype	Comments	References
*wadB*	Glycosyltransferase involved in the synthesis of *Brucella* LPS core lateral branch	S-LPS with core defect	*B. abortus* mutants shown to be attenuated in mice	[30, 31]
*wadD*	Glycosyltransferase involved in the synthesis of *Brucella* LPS core lateral branch	S-LPS with core defect	*B. abortus* mutants shown to be attenuated in mice	[29]
*wbkF*	Undecaprenyl-glycosyltransferase priming bactoprenol for O-chain polymerization	R-LPS with complete core	*B. melitensis* mutants shown to be attenuated in mice	[17]
*wzm*	Permease of the ABC system translocating the O-chain to the periplasm	R-LPS with complete core	Build up cytoplasmic O-chain on the inner membrane. *B. melitensis* mutants shown to be attenuated in mice and to trigger antibodies to the O-chain	[17, 55]
*ppdK*	Pyruvate phosphate dikinase (PEP ↔ pyruvate)	S-LPS	Required for growth on gluconeogenic media *B. abortus* mutants are attenuated in mice	[33, 34]
*pckA*	Phosphoenolpyruvate carboxykinase (oxaloacetate → PEP)	S-LPS	Inactive in *B. abortus* but not in *B. suis* biovar 5. Together with *ppdK* is required for growth of *B. suis* biovar 5 in gluconeogenic media. *B. suis* biovar 5 mutated in both *ppdK* and *pckA* are attenuated in mice	[33, 34]

## Materials and methods

### Bacterial strains and growth conditions

The bacterial strains and plasmids used are listed in Additional file 1. We used *B. suis* bv2 CITA 198 (herein Bs2WT) because, although Bs2WT and the *B. suis* bv2 reference strain (*B. suis* bv2 Thomsen) have the same PCR-RFLP pattern [3], the former shows a virulence pattern in mice typical of *B. suis* bv2 field strains and the latter is attenuated (Additional file 2).

Bacteria were routinely grown either in standard tryptic soy broth (TSB; Scharlau, Barcelona, Spain) or TSA (TSB supplemented with agar [Pronadisa, Laboratorios Conda, Spain]) at 37 °C. For the studies in mice, vaccines and challenge strains were grown on Blood Agar Base (BAB; Oxoid, UK). When needed, media were supplemented with 5% sucrose, diaminopimelic acid (DAP [Sigma]; 1 mM), 0.2% activated charcoal, kanamycin (Km) at 50 µg/mL or at 35 µg/mL, ampicillin (Amp) at 100 µg/mL and/or chloramphenicol (Cm) at 20 µg/mL (all from Sigma). The lactate-glutamate-glycerol-vitamins synthetic medium of Gerhardt’s [35] was supplemented with 1 mM methionine (mGSM) (this amino acid is required for growth of some *Brucella* strains in synthetic media [32] including Bs2WT (Zúñiga-Ripa, unpublished observations). All strains were stored at − 80 °C in skimmed milk (Scharlau, Barcelona, Spain) or in TSB supplemented with 0.5% yeast extract (Pronadisa, Laboratorios Conda, Spain) (TYSB) and 7% dimethylsulfoxide.

### Sequence analyses and DNA manipulation

Genomic sequences of *B. suis* bv2 Thomsen (ATCC 23445 or NCBI:txid470137), *B. melitensis* 16M (ATCC 23456 or NCBI:txid224914) and *B. abortus* 2308 (NCBI:txid359391) were obtained from databases at the National Center for Biotechnology Information (NCBI) or Kyoto Encyclopedia of Genes and Genomes (KEGG: https://www.genome.jp/kegg/). Since the genomic sequence of Bs2WT was not available, the ORFs of interest were PCR amplified and sequenced by “Servicio de Secuenciación del Centro de Investigación Médica Aplicada” (CIMA, Pamplona, Spain). Sequence alignments were performed with Clustal Omega (https://www.ebi.ac.uk/Tools/msa/clustalo/).

Plasmids and chromosomal DNA were extracted with QIAprep^®^ Spin Miniprep Kit and QIAamp^®^ DNA Miniprep Kit (Qiagen GmbH, Hilden, Germany). When needed, DNA was purified from agarose gels using a QIAquick^®^ Gel Extraction Kit (Qiagen GmbH, Hilden, Germany). Primers were designed using Primer 3 input (http://primer3.ut.ee/) and synthesized by Sigma-Genosys Ltd (Haverhill, United Kingdom).

### Mutant construction

We obtained Bs2Δ*wadB*, Bs2Δ*wadD*, Bs2Δ*ppdK*, Bs2Δ*ppdK*Δ*pckA*, Bs2Δ*ppdK*Δ*wadB* and Bs2Δ*ppdK*Δ*wadD* (all with O-chain) and Bs2Δ*wbkF* and Bs2Δ*wzm* (R; O-chain defective) by in-frame deletion of the corresponding genes (Table 1 and Additional file 1). For the construction of Bs2Δ*wadB*, Bs2Δ*wadD* and Bs2Δ*ppdK,* we used the suicide plasmids previously shown to generate in-frame deletions in *B. abortus* (the sequences where oligonucleotides hybridize are identical in Bs2WT) [29, 30, 33, 34]. To this end, the plasmids were extracted from *E. coli* TOP10F´ and transformed into *E. coli* β2150, a diaminopimelic acid (DAP) auxotrophic donor strain [36]. For Bs2Δ*wadB* construction, the suicide plasmid pJQKΔ*wadB* [30] was introduced in Bs2WT by tri-parental mating with conjugative *E. coli* β2150-pJQKΔ*wadB* and *E. coli* β2150-pRK2013 as helper strain [37]. The first recombination event was selected by Km resistance and DAP independence and confirmed by PCR. The allelic exchange by double recombination was selected for growth on sucrose and Km sensitivity. The same strategy was followed to construct Bs2Δ*wadD* and Bs2Δ*ppdK* using suicide plasmids pJQKΔ*wadD* [29] and pJQKΔ*ppdK* [34]. The double mutants Bs2Δ*ppdK*Δ*wadB* and Bs2Δ*ppdK*Δ*wadD* were constructed by deletion of *wadB* or *wadD* in Bs2Δ*ppdK* as described above. The double mutant Bs2Δ*ppdK*Δ*pckA* was constructed by deleting *pckA* in Bs2Δ*ppdK* using plasmid pJQKΔ*pckA* [34]. The loss of the plasmid concomitant with the gene deletion in each of these mutants was confirmed by PCR with the corresponding oligonucleotides (see Additional file 3).

For the construction of Bs2Δ*wzm*, we first generated two PCR fragments: oligonucleotides *wzm*-F1 and *wzm*-R2 (Additional file 4) were used to amplify a 484 bp fragment including codons 1 to 31 of BMEI1415, as well as 390 bp upstream of the start codon. Oligonucleotides *wzm*-F3 and *wzm*-R4 (Additional file 4) were used to amplify a 447 bp fragment including codons 247 to 265 of BMEI1415 and 385 bp downstream of the stop codon. These fragments were ligated by overlapping PCR using *wzm*-F1 and *wzm*-R4 for amplification, and the complementary regions between R2 and F3 for overlapping. The resulting sequence, containing the *wzm* deletion allele, was cloned into pCR2.1 (Invitrogen) to generate plasmid pCR2.1Δ*wzm,* sequenced to ensure the maintenance of the reading frame, subcloned into the *Bam*HI and *Xba*I sites of the suicide plasmid pJQK [38] and transformed into competent *E. coli* β2150. The resulting suicide pJQK-derived plasmid (pJQKΔ*wzm*) was introduced in Bs2WT by conjugation following the procedure described above. Bs2Δ*wbkF* was constructed following a similar strategy using oligonucleotides *wbkF*-F1 and *wbkF*-R2 (Additional file 4) that amplified a 448 bp fragment (including codons 1 to 19 of BMEI1426 as well as 390 bp upstream of the BMEI1426 start codon) and oligonucleotides *wbkF*-F3 and *wbkF*-R4 (Additional file 4) that amplified a 505 bp fragment (including codons 301 to 335 of BMEI1426 and 398 bp downstream of the BMEI1426 stop codon). Both fragments were ligated, cloned into pCR2.1 to generate plasmid pCR2.1Δ*wbkF*, subcloned into pJQK (pJQKΔ*wbkF*) and transformed into competent *E. coli* β2150. After conjugation with Bs2WT, we performed PCRs (primers are described in Additional file 3) to screen the resulting colonies for the *wbkF* or *wzm* deletion.

We constructed a kanamycin resistant challenge strain (Bs2::Tn7Km^R^) to discriminate the challenge and vaccine strains in the mouse protection experiments (see below). For this, we inserted the pUC18R6KT-miniTn7Km vector into the Bs2WT chromosome by tetra-parental conjugation and selected the conjugants harboring this modified miniTn7 by plating onto TSA-DAP-Km. The construct (Bs2::Tn7Km^R^) was examined by PCR [39] for the correct insertion and orientation of the transposon between the *glmS* and *recG* genes [40, 41].

### Bacteriological typing

All strains were typed following established *Brucella* procedures [2]: i.e. colonial morphology, urease, agglutination with anti-A and anti-M monospecific sera recognizing the cognate epitopes in the O-chain of *Brucella* S-LPS, susceptibility to thionine, fuchsine and safranin dyes and sensitivity to Tb, Wb, Iz and R/C phages. S/R colony morphology was studied by the crystal violet dye exclusion test and by acriflavine agglutination [2].

### Growth curves

To obtain inocula preconditioned for growth in each test medium (TSB or mGSM), bacteria were first grown in 10 mL of TSB in a 50 mL flask at 37 °C with orbital shaking for 18 h. In the case of TSB growth curves, these exponentially growing bacteria were harvested by centrifugation, resuspended at an O.D._600 nm_ of 0.1 in the same medium and 200 µL/well aliquots were dispensed by triplicate in Bioscreen multi-well plates. In the case of mGSM growth curves, the exponentially growing bacteria in TSB were harvested by centrifugation, resuspended in 10 mL of mGSM at an optical density at 600 nm (O.D._600 nm_) of 0.1, and incubated at 37 °C with orbital shaking for 18 h. These preconditioned bacteria were harvested by centrifugation, resuspended at an O.D._600 nm_ of 0.1 in the same medium and 200 µL/well aliquots were dispensed by triplicate in Bioscreen multi-well plates. The plates were cultivated in a Bioscreen C (Lab Systems) apparatus with continuous shaking at 37 °C. Growth was monitored spectrophotometrically at 420–580 nm every 30 min over a 60 h-period. All experiments were performed in triplicate (biological replicates). Wells containing sterile medium were used as negative controls in all experiments.

### LPS characterization

LPS from Bs2WT and mutants in LPS genes (Table 1) were extracted by the proteinase K sodium dodecyl sulfate (SDS) protocol [42], an extraction procedure valid for S and R *Brucella* [17, 43]. Briefly, phenol-inactivated bacteria (grown in TSB) were centrifuged (20 min at 6 500 × *g*) and washed with saline. The cells were suspended in 2% SDS-60 mM Tris–HCl buffer (pH 6.8), heated at 100 °C for 10 min and treated with proteinase K (60 μL of a 2.5 mg/mL stock in Tris–HCl per each mL of suspension) for 3 h at 55 °C and overnight at room temperature. Then, samples were centrifuged (30 min at 20 000 × *g*) and the supernatants precipitated with 3 volumes of methanol containing 1% sodium acetate-saturated methanol at -20 °C for 60 min. The precipitates were collected by centrifugation (15 min at 5 000 × *g*), and resuspended in 10 mL of distilled water, precipitated again with methanol and sedimented again by centrifugation. The pellets were resuspended in 3 mL of 60 mM Tris-HCl (pH 6.8), digested once with nucleases [20 µL/mL each of DNase and RNase stock solutions at 0.5 mg/mL in Tris–HCl (MP Biomedicals and Sigma-Aldrich, respectively)] at 37 °C for 30 min and treated again with proteinase K (60 µL/mL, 3 h at 55 °C). Finally, the LPS were harvested by centrifugation (15 min at 5 000 × *g*), suspended in 1 mL of distilled water and stored at − 20 °C.

For LPS characterization, LPS samples were analyzed in 15% polyacrylamide gels (37.5:1 acrylamide/methylene-bisacrylamide ratio) in Tris-HCl-glycine and revealed by the periodate-alkaline silver method [44]. For western blots, gels were electro-transferred onto nitrocellulose sheets (Amersham-GE Healthcare Life Scientific, Germany; 0.45 µm pore) using 25 mM Tris, 192 mM glycine (pH 8.3) and 20% (vol/vol) methanol in a Trans-Blot Semi-Dry Transfer Cell (Bio-Rad) at a constant voltage of 8 V and 200 mA for 30 min. Membranes were blocked with 5% skimmed milk in phosphate buffered saline (PBS; 10 mM phosphate 140 mM NaCl, 2.7 mM KCl [pH 7.4]; Medicago, Sweden) with 0.05% Tween 20 (PBS-T) overnight at 4 °C, and then washed with the same buffer. Membranes were then incubated with primary antibody diluted 1:500 in PBS-T overnight at room temperature, washed with PBS-T and bound immunoglobulins were detected with peroxidase labeled protein G and 4-chlorine-1-naphtol-H_2_O_2_. Monoclonal antibody A68-24G12/A08 from the ascitic fluid of an infected mouse, which recognizes core epitopes [45], and a serum of a rabbit infected with *B. melitensis* 16M and bled 45 days later were used.

### Mouse experiments

Seven-week-old female BALB/c mice (ENVIGO, Harlan) were lodged in cages in a BSL-3 facility (ES/31-2010-000132) with water and food ad libitum for 2 weeks before and during the experiments in accordance with the current European (directive 86/609/EEC) and Spanish (RD 53/2013) legislations.

For virulence assessment, groups of 5 mice per time-point were inoculated with each mutant (Bs2Δ*wadB*, Bs2Δ*wadD,* Bs2Δ*ppdK*, Bs2Δ*ppdK*Δ*wadB,* Bs2Δ*ppdK*Δ*wadD*, Bs2Δ*wzm* and Bs2Δ*wbkF*) or Bs2WT. Inocula were prepared harvesting BAB grown bacteria in sterile buffered saline (BSS; 0.85% NaCl, 0.1% KH_2_PO_4_, 0.2% K_2_HPO_4_; pH 6.85) and adjusting the bacterial suspension to 1 × 10^9^ CFU/mL (inoculum used for 1 × 10^8^ CFU doses in the immunization with R vaccines). This suspension was then diluted to 1 × 10^6^ CFU/mL to prepare the inocula used for S vaccines (1 × 10^5^ CFU doses). Mice were inoculated intraperitoneally (IP) with 0.1 mL of each inoculum and the exact dose was determined retrospectively on triplicate BAB plates. At several intervals after inoculation, mice were euthanized, the spleens were aseptically removed, individually weighed, homogenized in 9 volumes of BSS and serial tenfold dilutions plated by triplicate on BAB plates. After 5 days at 37 °C, CFU were counted, colonies checked by the crystal violet exclusion test and the identity confirmed by PCR. The data (mean CFU/spleen) were normalized by logarithmic transformation, and mean log_10_ CFU/spleen values and standard deviations (SD) calculated for plotting and statistical comparison by one-way ANOVA and Fisher's Protected Least Significant Differences (PLSD) tests.

To determine the protective efficacy, groups of 5 BALB/c mice were inoculated subcutaneously (SC) with 10^5^ CFU/mouse of Bs2Δ*ppdK*, Bs2Δ*wadB* and Bs2Δ*ppdK*Δ*wadB* or 10^8^ CFU/mouse of Bs2Δ*wbkF* and Bs2Δ*wzm*. The Rev1 reference vaccine (10^5^ CFU/mouse) or BSS inoculated mice were used as effective-vaccine and unvaccinated controls, respectively. Four weeks after vaccination, all animals were challenged IP with 10^5^ CFU of the virulent Bs2::Tn7Km^R^ strain. After 2 weeks, CFU/spleen numbers of the challenge strain were determined by plating on BAB Km. Values of residual vaccine were also calculated by subtracting CFU numbers on BAB Km to those obtained on BAB. Results were expressed as the mean log_10_ CFU/spleen ± SD (n = 5) and statistical comparisons between vaccines and controls for challenge strain values were made using ANOVA and PLSD tests. The virulence of the Bs2::Tn7Km^R^ challenge strain used in this work was verified in a previous experiment in BALB/c mice (n = 5) by IP infection (10^5^ CFU/mouse) and CFU/spleen counting 2 and 6 weeks later. This strain showed identical CFU in BAB-S and BAB-Km, and virulence pattern identical to that of Bs2WT (not shown).

## Results

### Construction and characterization of *B. suis* biovar 2 LPS mutants

We first confirmed the presence of the orthologues of *B. abortus wadB* and *wadD* in *B. suis* bv2 using the complete sequence of the genome of *B. suis* bv2 Thomsen available in databases. Then, as this strain was not suitable for determining the actual degree of attenuation of the mutants (see “Materials and methods” and Additional file 2), we amplified and sequenced the corresponding regions in the genome of Bs2WT, a virulent strain (Additional file 2). We found that these two ORFs were highly conserved in Bs2WT and, accordingly, we obtained the non-polar mutants Bs2Δ*wadB* and Bs2Δ*wadD* using the tools developed before for *B. abortus* (see Table 1 and Additional file 1).

**Figure 2:**
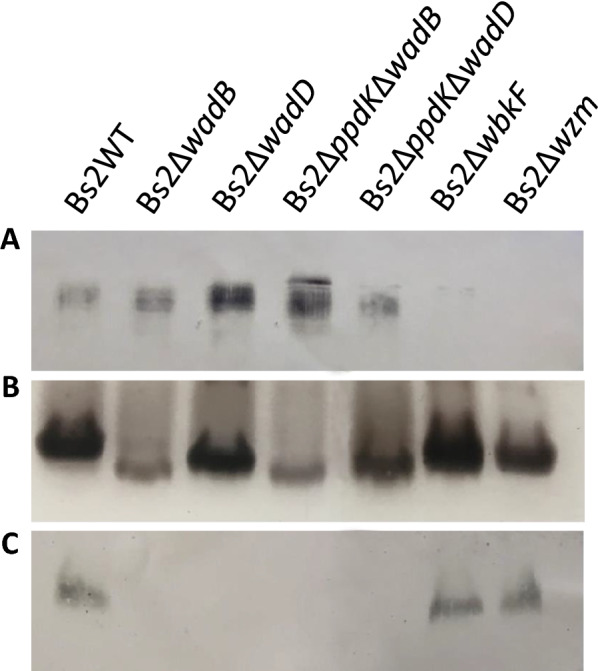
**LPS defects generated in*B. suis*bv2 by mutation of*wadB***, ***wadD***, ***wbkF*and*wzm***. **A** Western blot analysis of SDS-proteinase K extracts with an anti-S *Brucella* serum (rabbit infected with *B. melitensis* 16M). **B** SDS-PAGE and silver staining of SDS-proteinase K extracts. **C** Western blot analysis of SDS-proteinase K extracts with monoclonal anti-core antibody A68-24G12/A08.

Bs2Δ*wadB* and Bs2Δ*wadD* were identical to the Bs2WT parental strain in growth rates in TSB and mGSM (data not shown) as well as in urease production, sensitivity to thionine, fuchsine and safranin dyes, and in agglutination with the monospecific A but not with the monospecific M sera (Additional file 5). This last result indicates that the O-chain is conserved in the mutants as predicted by the function assigned to WadB and WadD (Table 1 and Figure 1A–B), and this was also confirmed by western blot analysis (Figure 2A). Analysis of the core region showed the predicted (Table 1) small molecular weight shift (Figure 2B) indicative of the lack of some sugars in the core lateral branch (Figure 1B) and the subsequent defect in the core epitope recognized by monoclonal A68-24G12/A08 (Figure 2C). Altogether, these results show that Bs2Δ*wadB* and Bs2Δ*wadD* are homologous in LPS structure to their *B. abortus* counterparts [29, 30].

Following the same genomic approach, we also constructed R-LPS mutants. First, we identified the O-chain primase (*wbkF*) and O-chain translocation (*wzm*) genes (Table 1) in *B. suis* bv2 Thomsen and then we confirmed by amplification and sequence analysis that they were conserved in Bs2WT. Based on this, we constructed the corresponding in frame-deletion mutants and confirmed the R phenotype by a negative result in the crystal violet exclusion test, a positive acriflavine agglutination and no agglutination with the monospecific anti-A and anti-M sera (that recognize O-chain epitopes with variable dominance depending on the S *Brucella* species and biovar). Although both mutants were resistant to phage R/C (Additional file 5), this was not totally unexpected because this phage (originally obtained from a mixture of S specific *brucella* phages by *N*-methyl-*N*′-nitro-*N*-nitrosoguanidine treatment and serial propagation on *B. abortus*, *B. ovis* and *B. canis*) is known to display variable activity depending upon the *Brucella* species origin of the R mutant [46]. Further analyses by SDS-PAGE and western blot showed that these mutants carried a LPS that lacked reactivity with antibodies recognizing O-chain epitopes and that, in contrast to Bs2Δ*wadB* and Bs2Δ*wadD,* displayed mobility and anticore antibody reactivity similar to those of the R-LPS fraction of Bs2WT (Figure 2A–C). Therefore, these mutants carry a R-LPS with a complete core, as predicted (Table 1).

### Construction and characterization of *B. suis* biovar 2 metabolic mutants

**Figure 3:**
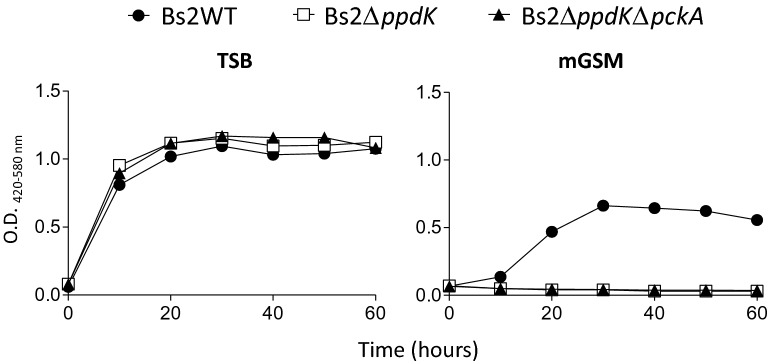
**Deletion of*ppdK*abolishes growth of*B. suis*bv2 in gluconeogenic (mGSM) but not in non-gluconeogenic (TSB) media.** Each point represents the mean ± standard error (error bars are within the size of symbols) values of technical triplicates. The experiment was repeated three times with similar results.

**Figure 4:**
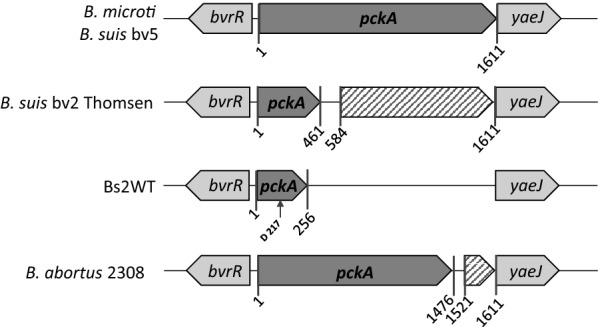
***B. suis*bv2 presents a SNP leading to a premature stop in*pckA***. The sites where premature stop codons occur are indicated with a vertical arrow. The SNP leading to premature stop in Bs2WT is indicated as D (for deletion). The functional *pckA* of *B. microti* and *B. suis* biovar 5, and the mutated *pckA* of *B. abortus* described previously are included as reference. In all brucellae, *pckA* is located upstream of the regulatory gene *bvrR. In Brucella* presenting an intact *pckA, yaeJ* (coding a ribosome-associated protein) is located immediately downstream of *pckA*, whereas in the sequenced *Brucella* presenting a mutated *pckA* a ORF (i.e.: a truncated PckA in *B. abortus* or a pseudogene in *B. suis* bv2 Thomsen) upstream of *yaeJ* is annotated (stripped arrows).

First, we searched for orthologues of *ppdK* and *pckA* (Table 1) in the *B. suis* bv2 Thomsen genome. We found that Thomsen *ppdK* is highly conserved with respect to *B. abortus* 2308 and *B. suis* 513 (biovar 5), two strains where its functionality has been shown (Table 1). Moreover, sequencing of the homologous region confirmed its conservation in Bs2WT. Therefore, we constructed a Bs2Δ*ppdK* in-frame deletion mutant of Bs2WT and compared its growth in TSB and mGSM. As can be seen in Figure 3, deletion of *ppdK* abrogated growth in the latter medium but not in TSB, indicating that in 3C and 4C substrates the PEP (phosphoenolpyruvate) (and growth in gluconeogenic conditions) depends on PpdK (Figure 1C). Although in *B. suis* biovar 5, PEP [33] can result also from the activity of PckA in gluconeogenic medium (Figure 1C), this observation suggested us that PckA is not functional in Bs2WT, a phenotype we already described for *B. abortus* [34]. In fact, the complete sequence of *B. suis* bv2 Thomsen shows a premature stop codon in *pckA* (Figure 4) not present in *B. suis* biovar 5 or *B. microti* [33]. A premature stop codon also occurs in *pckA* in all *B. abortus* and *B. melitensis* sequenced strains. However, while in these species the stop codon is located at position 1474–1476, in *B. suis* bv2 Thomsen it is located at position 492–494 (Figure 4 and Additional file 6). Upon sequencing, we observed that the Bs2WT also displayed a truncated version of *pckA* that interestingly resulted from a deletion at position 217 generating a premature stop codon at position 254–256, thus different from those in *B. abortus*, *B. melitensis* or *B. suis* bv2 Thomsen (Figure 4). Remarkably, a database search in KEGG showed that the same mutation found in *B. suis* bv2 Thomsen is present in the genome of all *B. suis* bv2 strains sequenced thus far. Indeed, a double mutant Bs2Δ*ppdK*Δ*pckA* showed the same inability to grow on mGSM as Bs2Δ*ppdK* (Figure 3). These findings strongly suggest that the lack of activity of PckA observed in *B. abortus* [33] is extended to at least *B. suis* bv2.

### *B. suis* vaccine candidates display different profiles of attenuation in mice

**Figure 5:**
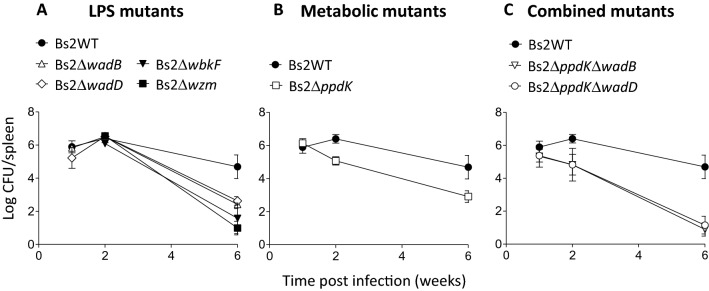
***B. suis*vaccine candidates display different profiles of multiplication in mouse spleen.** Multiplication profile of **A** LPS mutants, **B***ppdK* metabolic mutant and **C** combined mutants. CFUs in spleen of infected BALB/c mice were counted after SC inoculation with 10^5^ CFU/mouse of Bs2Δ*ppdK*, Bs2Δ*wadB*, Bs2Δ*wadD*, Bs2Δ*ppdK*Δ*wadB* Bs2Δ*ppdK*Δ*wadD,* or 10^8^ CFU/mouse of Bs2Δ*wbkF* and Bs2Δ*wzm.*

We evaluated the colonization and persistence of Bs2WT mutants in the spleen of mice by IP inoculation with 1 × 10^8^ and 1 × 10^5^ CFU/mouse of mutants with R and S LPS, respectively (Figure 5). The CFU/spleen values of all LPS mutants were similar to those of Bs2WT at post-infection week 2. However, at post-infection week 6, the CFU/spleen of all these mutants were significantly lower (p < 0.005) than those of Bs2WT but with different degrees of attenuation (Figure 5A). The Bs2Δ*wbkF* and Bs2Δ*wzm* R mutants showed numbers of CFU/spleen significantly lower (p < 0.05 and p < 0.0005) than those in the spleens of Bs2Δ*wadB* or Bs2Δ*wadD* inoculated mice, which were not different from each other. In contrast to the LPS mutants, attenuation generated by *ppdK* dysfunction was observed (p < 0.05) already after 2 weeks (Figure 5B). Interestingly, at week 6 the *ppdK* mutant remained in the spleen at levels higher than those of the R mutants (p < 0.005) and similar to those of the LPS core mutants Bs2Δ*wadB* and Bs2Δ*wadD* (Figure 5B). At the doses used, all mutants induced more splenomegaly than Bs2WT at the peak of the infection, consistent with a higher stimulation of immunity that could in part account for their attenuation (Additional file 7). The above-summarized differences in attenuation profiles prompted us to construct vaccine candidates by combining core and metabolic defects. We constructed mutants carrying in-frame deletions in *ppdK* and *wadB* (Bs2Δ*ppdK*Δ*wad*B) or in *ppdK* and *wadD* (Bs2Δ*ppdK*Δ*wadD*). Growth in either TSB or mGSM was identical to that obtained with Bs2Δ*ppdK* (not shown). As expected, the double mutants showed a LPS profile similar to those of Bs2Δ*wadB* and Bs2Δ*wadD* (Figure 2). Like Bs2Δ*ppdK*, Bs2Δ*ppdK*Δ*wad*B and Bs2Δ*ppdK*Δ*wadD* were attenuated (p < 0.005) at post-infection week 2 and more attenuated than the corresponding single mutants (p < 0.0005) at post-infection week 6 (Figure 5C), consistent with an additive effect of the mutations and the multiplication defect observed for the *ppdK* mutant 2 weeks after infection (Figure 5B). As expected from the lower bacteria load, splenomegaly was reduced as compared to that observed for the single mutants (Additional file 7).

### Protection in mice

Based on the above-summarized results, we selected mutants representing the different multiplication profiles for a protection experiment in mice: Bs2Δ*ppdK*, Bs2Δ*wadB*, Bs2Δ*ppdK*Δ*wad*B, Bs2Δ*wbkF* and Bs2Δ*wzm.* The protection efficacy of these mutants was compared with that conferred by Rev1 and mice inoculated with BSS as a control. As can be seen in Table 2, Rev1 (at 10^5^ CFU/mouse) and the five candidates (at 10^5^ or 10^8^ CFU/mouse for S or R phenotypes, respectively) provided similar levels of protection against Bs2WT.

**Table 2:** **Protection against virulent*B. suis*induced by LPS and metabolic mutants inoculated subcutaneously.**

Vaccine	Dose (CFU/mouse)	Mean ± SD of log_10_ CFU in spleen of	
		Bs2WT	Vaccine
Bs2Δ*wadB*	10^5^	3.97 ± 0.37^a,b^	2.61 ± 0.97
Bs2Δ*ppdk*	10^5^	3.65 ± 0.43^a,b^	2.39 ± 0.54
Bs2Δ*ppdk*Δ*wadB*	10^5^	4.12 ± 1.09^a,b^	2.68 ± 1.17
Bs2Δ*wbkF*	10^8^	3.33 ± 0.36^a,b^	2.45 ± 0.56
Bs2Δ*wzm*	10^8^	2.83 ± 1.88^a,b^	2.52 ± 1.50
Rev1	10^5^	3.68 ± 0.78^a^	2.43 ± 0.56
BSS	–	6.69 ± 0.13	

Statistical comparison (n = 5) of mean log_10_ Bs2WT CFU/spleen: ^a^p < 0.001 vs. BSS (unvaccinated) and ^b^p > 0.05 vs. Rev1.

## Discussion

Whereas vaccines against brucellosis that are effective in cattle, sheep and goats, were developed long ago, this is not the case of a vaccine against swine brucellosis. In part, this may be due to the higher emphasis placed on ruminant brucellosis in industrialized countries as well as to the fact that outbreaks in pigs can be controlled by hygienic measures and stamping out under the conditions prevailing in these countries [4]. Altogether, these circumstances have made less urgent the development of a *B. suis* vaccine. However, there is a reemergence of swine brucellosis in continental Europe (caused by *B. suis* bv 2) mainly because of the increase in production systems in which interaction with raising populations of wild boars becomes unavoidable and depopulation unfeasible. Although antibiotic treatment combined with culling may work for *B. suis* bv 2 outbreaks [47], this strategy cannot be generally applied and the use of antibiotics for these purposes is against WHO recommendations [48]. Moreover, it has been pointed out [4] that the huge change in pig breeding conditions taking place in many emerging economies and the fact that *B. suis* biovars 1 and 3 are highly zoonotic, create conditions where an effective *B. suis* vaccine would become very useful. Another reason for controlling the disease in pigs is that they can transmit the infection to cattle and that cows can excrete *B. suis* in milk multiplying the zoonotic risk [49].

Live attenuated rather than subcellular vaccines are of choice for developing new brucellosis vaccines [50], they are less costly to manufacture and, most important, mimic critical *Brucella* properties (cell invasion, trafficking, and suitable antigen presentation) needed to induce protective immunity. However, new brucellosis live vaccines should circumvent drawbacks posed by those currently used in ruminants (S19, Rev1 and RB51), particularly their potential to infect humans and resistance to antibiotics used in human brucellosis. We chose the LPS as a first target for developing an attenuated vaccine because this molecule is a key virulence factor of brucellae as well as an immunodominant antigen [51], and our current understanding of its genetics makes it feasible to obtain LPS mutants with a variety of phenotypes.

Concerning lipid A, the only *Brucella* lipid A mutant affected in the modified pathway (*lptA*) is not attenuated in mice [52], and no mutants in the conserved Raetz pathway [53] have been described in *Brucella,* possibly because they are not viable (Conde-Álvarez, Iriarte and Letesson, unpublished). On the other hand, the core oligosaccharide and the O-chain genes are amenable to mutation. Previous investigations show that the structure of the LPS of at least *B. abortus* and *B. melitensis* is such that mutations in core glycosyltransferases can generate either S (i.e. O-chain bearing) or R (i.e. O-chain lacking) phenotypes (Figure 1B) [18, 29, 30, 54]. In line with previous observations in *B. melitensis* and *B. abortus*, we found that *B. suis wadB* and *wadD* mutants carry a core oligosaccharide defect that generates attenuation while not disturbing the O-chain. Therefore, both mutants are a first type of LPS vaccine candidates, and as they display similar attenuation, we chose Bs2Δ*wadB* for the protection experiments. It has been repeatedly shown that R mutants (i.e. lacking an O-chain linked to the core) of S brucellae are also attenuated and thus represent live vaccine candidates [19]. However, R mutants display various degrees of attenuation depending on the extent of core defect, and work with *B. abortus* suggests that maintaining an intact core is necessary for an optimal R vaccine candidate [55]. As depicted in Figure 1A, B, there are two types of R mutants with a complete core: those devoid of O-chain (mutants in *wboA*, *wboB*, *wbkA*, *wbkE* or *wbkF*) and those carrying cytoplasmic O-chain precursors (mutants in *wadA* and *wzm/wzt*) [17, 56]. In this work, we tested a mutant of each kind (Bs2Δ*wbkF* and Bs2Δ*wzm*) and we found that they were cleared faster than Bs2Δ*wadB* or Bs2Δ*wadD* despite being inoculated at a dose 1000 fold higher, a result consistent with similar comparisons in *B. abortus* and *B. melitensis*. Attenuation was similar for Bs2Δ*wbkF* and Bs2Δ*wzm* but since the latter carries O-chain precursors that have been reported to improve protection in an R background (see below), we tested both.

Despite their different LPS defects and degree of attenuation, we obtained similar protection with Bs2Δ*wadB*, Bs2Δ*wbkF*, Bs2Δ*wzm* and the Rev1 control. Although these results demonstrate protective immunity in mice, they have to be understood in the context of the experimental conditions. Indeed, because of the high costs and long-time span of experiments in natural hosts, mice have been used as a first step in the analyses of brucellosis vaccines (reviewed in [57]). However, it is important to keep in mind that the BALB/c mouse model commonly used for these purposes was originally developed for the biological control of master seeds of S19 and Rev1, and that results with vaccine candidates are affected by at least, dose of vaccine and challenge, challenge strain, and time span between vaccination and challenge, so that control by inclusion of S19 or Rev1 is critically important [57]. In this work, we have followed the recommended procedures for all these parameters [57], and the only bias in our comparisons is in the use of different doses, 10^8^ CFU for the two R vaccines and 10^5^ CFU for Bs2Δ*wadB* (or Bs2Δ*ppdK*, see below) and Rev1. These doses were chosen because of the consistent observation that R vaccine candidates require 100 to 1000 fold higher doses than S19 or Rev1 to produce a comparable protection in mice [17, 58, 59]. However, controlled experiments in cattle or sheep with *B. abortus* or *B. melitensis* R mutants show that even with an increase in the dose they are not as effective as S19 or Rev1 [18, 19], indicating that *B. abortus* or *B. melitensis* R strains induce comparatively lower protection than S vaccines. To know whether this limitation also applies to their *B. suis* homologues requires controlled experiments in swine. This is worth studying because an optimal R vaccine should not interfere in diagnostic tests that use whole S *Brucella* cells (rose bengal and complement fixation) [60]. However, the presence of O-chain precursors in *Brucella* R mutants has been shown to trigger antibodies interfering in all tests [60] and therefore, as the Bs2Δ*wzm* did not yield better results, Bs2Δ*wbkF* is a better candidate for a *B. suis* R vaccine. Nevertheless, since all R vaccines interfere in other tests (indirect and competitive ELISA, fluorescence polarization assay, etc.) [60] that are widely used, it is not immediately clear whether Bs2Δ*wbkF* would be advantageous when compared with a S candidate like Bs2Δ*wadB* that might result in better protection in the natural host.

We also investigated a metabolic mutant. The rational for this strategy is to reduce multiplication by hampering *B. suis* central C metabolism in pathways connecting PEP and TCA (tricarboxylic acid cycle) (Figure 1C). We have shown before that while both PckA and PpdK are involved in these steps in *B. suis* biovar 5, only PpdK is active in *B. abortus* [33]. Interestingly, the same mutation disabling *B. abortus* PckA is present in *B. melitensis* (see above and [33]), and we report here that *B. suis* bv2 *pckA* is also mutated albeit in a different position. Taken together, these observations strongly suggest that the function of PckA is irrelevant with regards to virulence in ruminants and swine. On the contrary, the single Bs2Δ*ppdK* mutant was attenuated, and this is reminiscent of the attenuation observed for single *ppdK* mutants of *B. abortus* in mice [34] and of *B. melitensis ppdK* in sheep (Zúñiga-Ripa, Conde-Álvarez, Iriarte, de Miguel, Arce-Gorvel, Gorvel, Moriyón, Blasco, and Muñoz, unpublished). Here, we also show that the attenuation generated by hampering C metabolism at the PEP-TCA connection could be useful in the development of brucellosis vaccines. Since at a 10^5^ CFU dose Bs2Δ*ppdK* conferred the same level of protection as Rev1, it is also an interesting vaccine candidate. Although S vaccines generate interference in serodiagnosis, the use of the conjunctival route has been shown to minimize the problem in ruminants [20, 60] and it could also be effective in pigs.

The literature shows that the reduction in mouse spleen colonization has been reported previously for several *B. suis* vaccine candidates. Nasal immunization with a recombinant adhesin (BtaF) in cyclic-di-AMP protected mice against *B. suis* bv1 administered intragastrically but not when challenged intratracheally [61]. However, the protocol of immunization and costs indicate that this vaccine when improved with BtaF synergic antigens [61] would be more suitable for human use. Strain VTRS1, an R *wboA* mutant of *B. suis* biovar 4 [22], when administered intravenously (IV), was more efficacious against *B. suis* bv4 than RB51 administered IP. However, the reference S19 vaccine (IV) yielded better results [22]. Later, a VTRS1 homologue (VTRS2) created in a *B. suis* biovar 1 (strain 1330) over-expressing the potentially immunocontraceptive multimeric GnRH protein was developed but this construct did not afford protection against *B. suis* 1330, even after a booster administration [23]. Also following the R vaccine approach, a *B. suis* biovar 1 R mutant in *pgm* (Figure 1B) reduced spleen colonization in mice challenged with *B. suis* 1330, but no reference vaccine control was included [21]. Also, *pgm* mutants carry an incomplete core and are defective in the synthesis of periplasmic β-glucans, a pleiotropic effect that may explain why comparison with *B. melitensis* showed that they were less protective than mutants with a full core [17].

RB51 has been manipulated in attempts to improve the poor performance observed in the above-summarized experiment. A first study [25] constructed derivatives over-expressing WboA (disrupted by IS711 in RB51) (Figure 1A, B illustrate the role WboA in LPS synthesis), the L7/L12 ribosomal protein and the Cu/Zn superoxide dismutase (SOD) in a *leuB* mutant of RB51 using plasmid pNS4 (which is stable because it complements the *leuB* defect) [62]. The best construct (RB51*leuB*/SOD/WboA) administered IP reduced *B. suis* 1330 spleen colonization but no reference vaccine control was included. In a more recent work [26], the expression of O-chain was further enhanced by over-expressing *wboA* and *wbkF*. The construct (RB51*WboAKF*) provided protection against *B. suis* 1330 similar to that of the *wboA* mutated R *B. suis* VTRS1 [26]), and improved VTRS1 when administered at a higher dose. However, no reference vaccine was included in the experiments of protection against *B. suis*. The rational for restoring the O-chain in RB51 is not obvious because of the rifampicin resistance and infectivity for humans of RB51 [27] and also because it offsets the main potential advantage of R mutants as vaccines (i.e. not triggering antibodies to the S-LPS).

Some vaccine candidates developed in a *B. neotomae* background have also been tested against *B. suis* in mice. Moustafa et al. [63] investigated gamma-inactivated *B. neotomae*, and two recombinants over-expressing Bp26 and SOD, administered twice IP conferred protection against *B. suis* 1330. This study did not include a control with a standard vaccine seemingly because the main aim was to develop a human vaccine (in animals a booster immunization is most often difficult to implement). Similarly, because of the potential advantages of R vaccines with regards to some serological tests, a *B. neotomae wboA* mutant (strain BNW) has been tested [24]. However, it is to be noted that, although *B. neotomae* is currently accepted as a BSL2 *Brucella*, there is increasing evidence of its zoonotic potential [64] and wide range [65].

The vaccine candidates described here afforded protection in mice similar to that obtained with Rev1 and, although the variety of experimental conditions make comparisons difficult, they seem to be in this regard similar or better than those in previous works. Indeed, optimal live brucellosis vaccines should show stable attenuation, lack antibiotic markers and be easy to discriminate from wild-type bacteria; moreover, they should not be infectious for humans. Since we generated attenuation by in-frame deletion, which makes the mutation irreversible and easy to detect by PCR, did not use antibiotic markers and circumvented the main issues related to infectiveness in humans by using *B. suis* bv2, these candidates seem to offer clear advantages. Indeed, live attenuated vaccines should have a time span of multiplication in the host long enough to elicit a protective immune response and be cleared soon enough to avoid abortions and excretion. Whether the multiplication and permanence in pigs of these attenuated candidates are adequate requires experiments in the natural host before they can be assessed for protection. In this regard, exploring the full potential of vaccines constructed in *B. suis* bv2 also requires investigation of their effectiveness against other *B. suis* biovars also infecting these domestic animals.

## Supplementary information

**Additional file 1. Bacterial strains and plasmids.**

**Additional file 2.*B. suis*1330 and*B. suis*CITA 198 (Bs2WT) but not*B. suis*bv2 Thomsen are virulent in mice.** Mice were inoculated intraperitoneally (IP) with 1 × 10^5^ CFU/mouse.

**Additional file 3. Primers and PCR products expected in mutant construction.**

**Additional file 4. Oligonucleotide sequences used for Bs2Δ*wzm*and Bs2Δ*wbkF*mutant constructions.**

**Additional file 5. Differential characteristics of species of the genus*Brucella*and mutants.**

**Additional file 6. Bs2WT*pckA*frameshift is in a position different from that in other*Brucella*species that carry a mutated PckA.** Alignment of PckA of *B. microti* and *B. suis* biovar 5, *B. melitensis* 16M and *B. abortus* 2308, *B. suis* bv2 Thomsen and Bs2WT. The amino acids differing are indicated in bold and shaded in gray.

**Additional file 7. Splenomegaly induced by the Bs2 mutants investigated.**
