# Supplementary material for: Development of attenuated live vaccine candidates against swine brucellosis in a non-zoonotic B. suis biovar 2 background
Source: Vet Res. 2020 Jul 23;51:92. doi: 10.1186/s13567-020-00815-8 (PMC7376850; doi:10.1186/s13567-020-00815-8)
Supplement: Supplementary file 1 — Additional file 1. Bacterial strains and plasmids. [file 13567_2020_815_MOESM1_ESM.docx]

**Additional file 1. Bacterial strains and plasmids**

| **Strains** | **Characteristics** | **Reference** |
| --- | --- | --- |
| ***Brucella suis*** |  |  |
| *B. suis* 1330 | *B. suis* biovar 1 reference strain; isolated from swine | ATCC 23444; CITA collection |
| *B. suis* Thomsen | *B. suis* biovar 2 reference strain; isolated from hare | ATCC 23445; CITA collection |
| Bs2WT | *B. suis* biovar 2 CITA 198; wild-type strain, isolated from wild boar; smooth LPS | CITA collection |
| Bs2Δ*wadB* | Bs2WT carrying an internal deletion in *wadB* gene (Δ_49-195_) | This work |
| Bs2Δ*wadD* | Bs2WT carrying an internal deletion in *wadD* gene (Δ_50-281_) | This work |
| Bs2Δ*ppdk* | Bs2WT carrying an internal deletion in *ppdK* gene (Δ_35-888_) | This work |
| Bs2Δ*ppdK*Δ*pckA* | Bs2WT carrying an internal deletion in *ppdK* (Δ_35-888_) and *pckA* (Δ_14-454_) genes | This work |
| Bs2Δ*ppdk*Δ*wadB* | Bs2WT carrying an internal deletion in *ppdK* and *wadB* genes | This work |
| Bs2Δ*ppdk*Δ*wadD* | Bs2WT carrying an internal deletion in *ppdK* and *wadD* genes | This work |
| Bs2Δ*wbkF* | Bs2WT carrying an internal deletion in *wbkF* gene (Δ_19-300_) | This work |
| Bs2Δ*wzm* | Bs2WT carrying an internal deletion in *wzm* gene (Δ_31-246_) | This work |
| Bs2::Tn7Km^R^ | Bs2WT with mini Tn7 transposon (pUC18R6KT-miniTn7T-Km). Challenge strain | This work |
| ***Escherichia coli*** |  |  |
| TOP10F´ | F-, lac/q Tn10 (Tetr), *mcrA* Δ(mrr-hsdRMS-mcrBC), 80*lacZ*ΔM15. Δl*acX*74 *recA*1alaD139 Δ (ara-leu)7697 *galU*, *galK*, *rpsL* and A1 *nupG* | Invitrogen |
| β2150 | F´lacZΔM15 *laclq* *pro*A+B+ *thr*B1004 *pro thi strA hsds ΔdapA::erm (Ermr) pir.*  *E. coli* deficient in the diaminopimelic acid (DAP) synthesis | [66]^1^ |
| SM10 λpir | *th*-1 *thr leu tonA lacY supE, recA*::RP4-2-Tc::Mu KmR (λpir). | [67]^1^ |
| **Plasmids** |  |  |
| pRK2013 | Helper vector containing *tra* and *mob* genes | [37] |
| pCR2.1 | Cloning vector, Km^R^ | Invitrogen |
| pJQK | Derivative plasmid of pJQ200KS+; Km^R^; Gm^S^ | [38] |
| pTNS2 | Plasmid expressing *tns*ABCD from P*lac.* ApR | [40] |
| pJQKΔ*wadB* | *Bam*HI-*Xba*I fragment from pYRI-1 (containing 570 bp of *B. abortus* chromosomal DNA with the *wadB* deletion allele) cloned into the corresponding sites of pJQKm (Internal code pYRI-2) | [30] |
| pJQKΔ*wadD* | *Xba*I fragment of 479 bp from *B. abortus* chromosomal DNA containing the *wadD* deletion allele cloned into the corresponding sites of pJQKm by *In-Fusion*® HD Cloning System (Internal code pMSB-34) | [29] |
| pJQKΔ*ppdK* | *Bam*HI-*Xba*I fragment from pMZI-1 (containing 508 bp of *B. abortus* chromosomal DNA containing the *ppdK* deletion allele) cloned into the corresponding sites of pJQKm (Internal code pMZI-2) | [34] |
| pJQKΔ*pckA* | *Bam*HI-*Xba*I fragment from pAZI-5 (containing 538 bp of *B. abortus* chromosomal DNA containing the *pckA* deletion allele) cloned into the corresponding sites of pJQKm (Internal code pAZI-6) | [34] |
| pCR2.1Δ*wbkF* | 953 bp of *B. melitensis* 16M chromosomal DNA containing the BMEI1426 deletion allele, generated by PCR and cloned into pCR2.1 (Internal code pRCI-16) | This work |
| pJQKΔ*wbkF* | *Bam*HI-*Xba*I fragment from pRCI-16 (containing 953 bp of *B. melitensis* 16M chromosomal DNA containing the *wbkF* deletion allele) cloned into the corresponding sites of pJQKm (Internal code pRCI-17) | This work |
| pCR2.1Δ*wzm* | 931 bp of *B. melitensis* 16M chromosomal DNA containing the BMEI1415 deletion allele, generated by PCR and cloned into pCR2.1 (Internal code pAZC-1) | This work |
| pJQKΔ*wzm* | *Bam*HI-*Xba*I fragment from pAZC-1 (containing 931 bp of *B. melitensis* 16M chromosomal DNA containing the *wzm* deletion allele) cloned into the corresponding sites of pJQKm (Internal code pAZC-2) | This work |
| pUC18 R6KT-miniTn7T-KmR | pUC18R6KT-miniTn7T-Km with Km cassette. Broad host-range mini-Tn7 vector | [41] |

^1^ Citations 66 and 67 referred below do not appear in the main list of the article:

[66] Dehio C, Meyer M (1997). Maintenance of broad-host-range incompatibility group P and group Q plasmids and transposition of Tn5 in Bartonella henselae following conjugal plasmid transfer from Escherichia coli. J Bacteriol. 179:538–540.

[67] Miller VL, Mekalanos JJ (1988) A novel suicide vector and its use in construction of insertion mutations: osmoregulation of outer membrane proteins and virulence determinants in *Vibrio cholerae* requires toxR. J Bacteriol. 70 (6): 2575-83.
