# Supplementary figures and images for: Development of attenuated live vaccine candidates against swine brucellosis in a non-zoonotic B. suis biovar 2 background
Source: Vet Res. 2020 Jul 23;51:92. doi: 10.1186/s13567-020-00815-8 (PMC7376850; doi:10.1186/s13567-020-00815-8)

## Slide 1
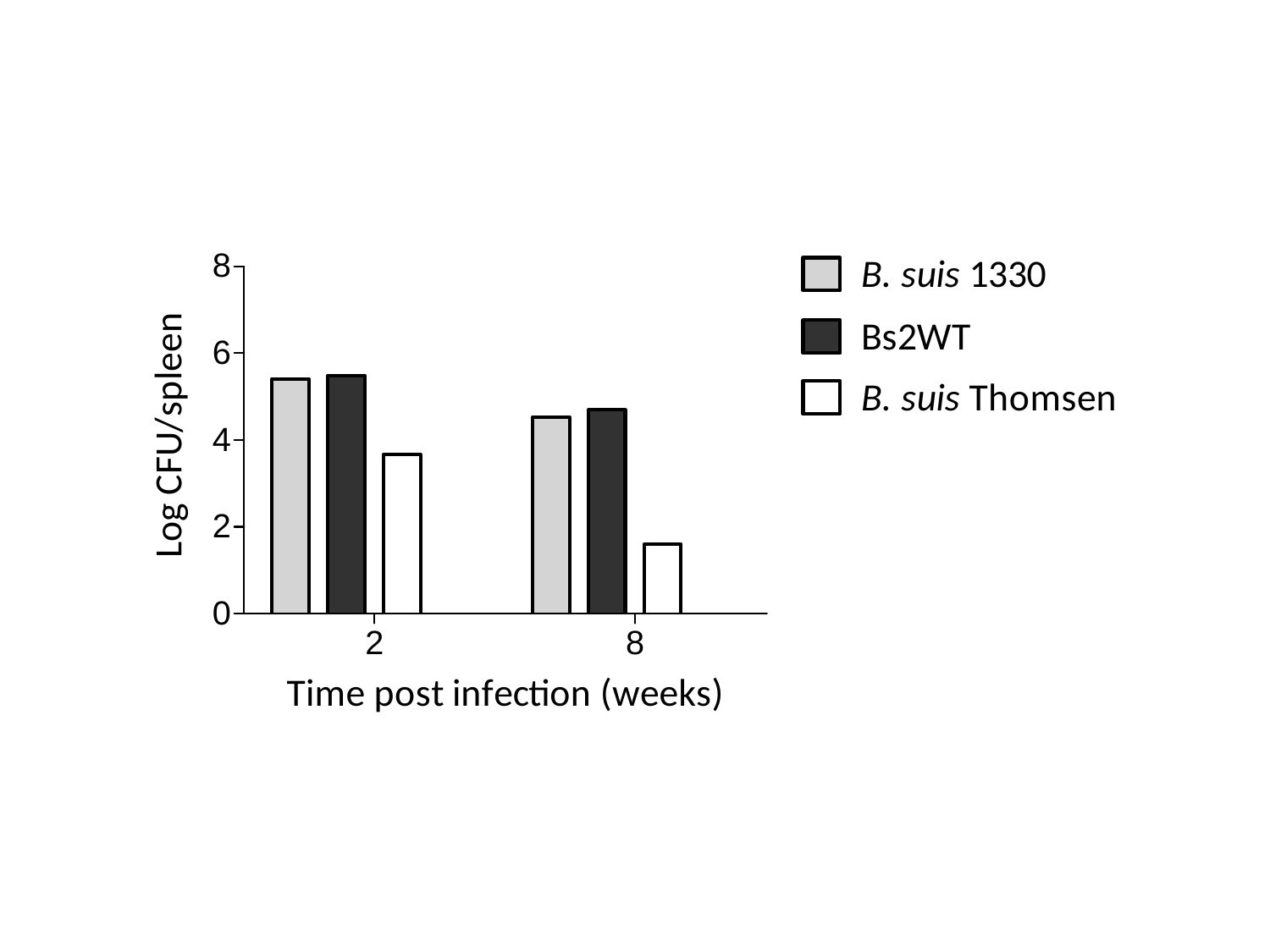

Supplement: Supplementary file 2 — Additional file 2.B. suis1330 andB. suisCITA 198 (Bs2WT) but notB. suisbv2 Thomsen are virulent in mice. Mice were inoculated intraperitoneally (IP) with 1 × 10^5^ CFU/mouse. [file 13567_2020_815_MOESM2_ESM.pptx]

## Slide 1
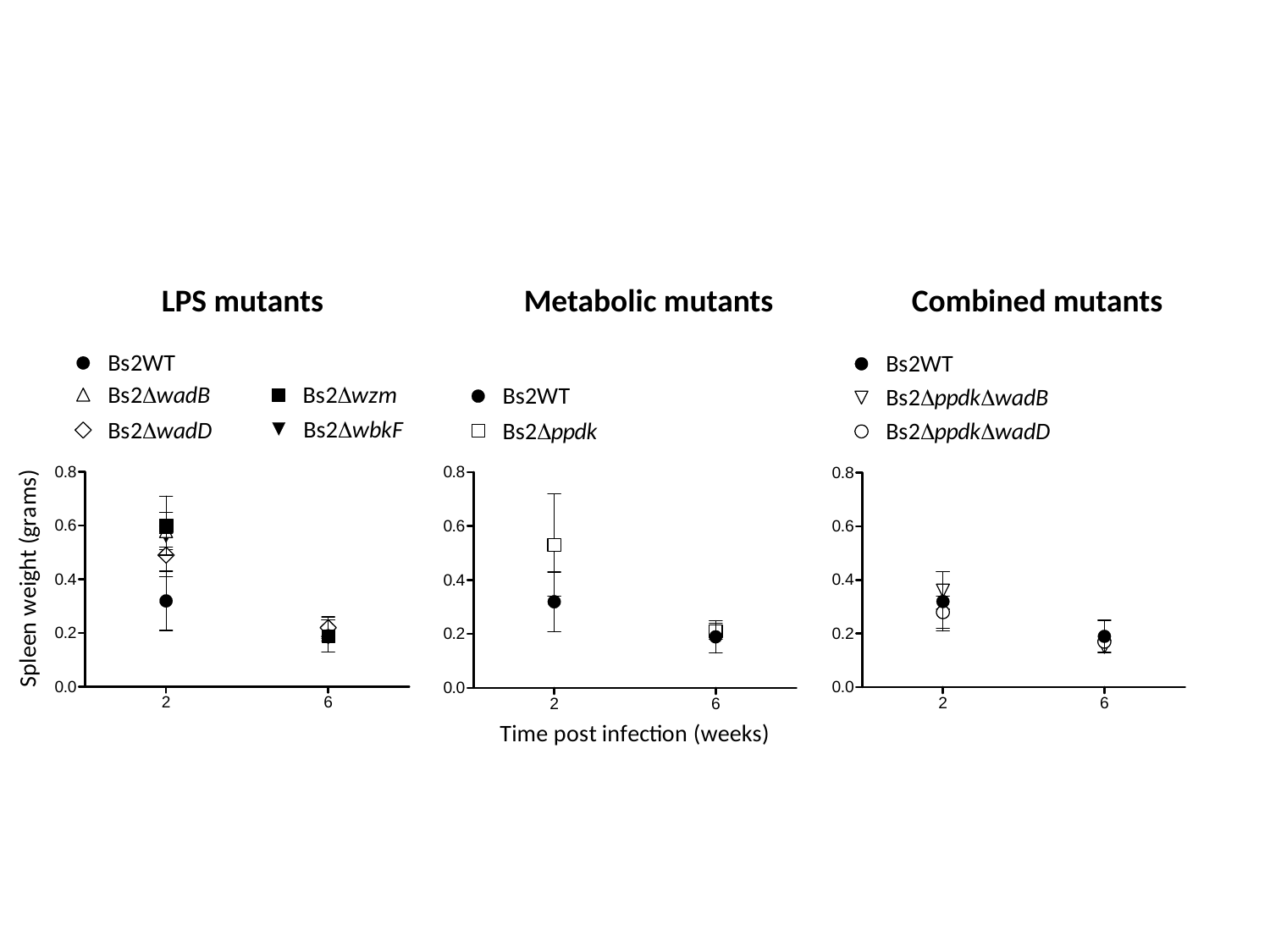

LPS mutants
Metabolic mutants
Combined mutants

Supplement: Supplementary file 7 — Additional file 7. Splenomegaly induced by the Bs2 mutants investigated. [file 13567_2020_815_MOESM7_ESM.pptx]
