# Supplementary material for: Development of attenuated live vaccine candidates against swine brucellosis in a non-zoonotic B. suis biovar 2 background
Source: Vet Res. 2020 Jul 23;51:92. doi: 10.1186/s13567-020-00815-8 (PMC7376850; doi:10.1186/s13567-020-00815-8)
Supplement: Supplementary file 3 — Additional file 3. Primers and PCR products expected in mutant construction. [file 13567_2020_815_MOESM3_ESM.docx]

**Additional file 3. Primers and PCR products expected in mutant construction**

| **Region amplified** | **Primers** | **Amplified fragment (bp)** | | **Reference** |
| --- | --- | --- | --- | --- |
|  |  | **In the mutant** | **In the sibling revertant strain** |  |
| *wadB* | *wadB*-F1 | 570 | 1011 | [30] |
|  | *wadB*-R4 |  |  |  |
| *wadD* | *wadD-*F1 | 479 | 1169 | [29] |
|  | *wadD*-R4 |  |  |  |
| *ppdK* | *ppdK*-F1 | 508 | 2983 | [34] |
|  | *ppdK*-R4 |  |  |  |
| *pckA* | *pckA*-F1 | 538 | 1864 | [34] |
|  | *pckA*-R4 |  |  |  |
| *wzm* | *wzm*-F1 | 931 | 1573 | This work |
|  | *wzm*-R4 |  |  |  |
| *wzm* | *wzm*-F1 | --- | 724 | This work |
|  | *wzm*-R5 |  |  |  |
| *wbkF* | *wbkF-*F1 | 953 | 1796 | This work |
|  | *wbkF-*R4 |  |  |  |
| *wbkF* | *wbkF-*F1 | --- | 680 | This work |
|  | *wbkF*-R6 |  |  |  |
