# Supplementary material for: Development of attenuated live vaccine candidates against swine brucellosis in a non-zoonotic B. suis biovar 2 background
Source: Vet Res. 2020 Jul 23;51:92. doi: 10.1186/s13567-020-00815-8 (PMC7376850; doi:10.1186/s13567-020-00815-8)
Supplement: Supplementary file 4 — Additional file 4. Oligonucleotide sequences used for Bs2Δwzmand Bs2ΔwbkFmutant constructions. [file 13567_2020_815_MOESM4_ESM.docx]

**Additional file 4. Oligonucleotide sequences used for Bs2Δ*wzm* and Bs2Δ*wbkF* mutant constructions.**

| **Primers** | **Sequence 5´- 3´** | **Used for** |
| --- | --- | --- |
| *wzm*-F1 | GCAAATTGAAATGGCAGATG | Overlapping PCR |
| *wzm*-R2 | AGCGCCCACGTAAATCAG | Overlapping PCR |
| *wzm*-F3 | CTGATTTACGTGGGCGCTTAACCTGCGTGGCAGTAGC | Overlapping PCR |
| *wzm*-R4 | ATGAAACGTGGCGTTAGTCC | Overlapping PCR |
| *wzm*-R5 | GCGTGTAAATTGCAAGAGGA | Verifying the *wzm* deletion |
| *wbkF-*F1 | TGGCTACGCCCCAACAAATC | Overlapping PCR |
| *wbkF-*R2 | AAGCCCAATCCCGCAGAGA | Overlapping PCR |
| *wbkF*-F3 | TCTCTGCGGGATTGGGCTTTTGCTGGCGCTGGATCATC | Overlapping PCR |
| *wbkF-*R4 | GACGCGCTATTTCATGTCCATC | Overlapping PCR |
| *wbkF*-R6 | TTCATAGCGGTCATCCAGTCC | Verifying the *wbkF* deletion |
|  |  |  |
