# Supplementary material for: Development of attenuated live vaccine candidates against swine brucellosis in a non-zoonotic B. suis biovar 2 background
Source: Vet Res. 2020 Jul 23;51:92. doi: 10.1186/s13567-020-00815-8 (PMC7376850; doi:10.1186/s13567-020-00815-8)
Supplement: Supplementary file 5 — Additional file 5. Differential characteristics of species of the genusBrucellaand mutants. [file 13567_2020_815_MOESM5_ESM.docx]

**Additional file 5. Differential characteristics of species of the genus *Brucella* and mutants.**

|  |  |  | **Thionine (µg/mL)**  with / without CO_2_ | | | **Fuchsin (µg/mL)**  with / without CO_2_ | | **Safranin (µg/mL)**  with / without CO_2_ | **Lysis by phages ^a^**  (RTD) ^b^ | | | |
| --- | --- | --- | --- | --- | --- | --- | --- | --- | --- | --- | --- | --- |
| **Strain** | **Urease activity** | **Serum anti  A / M** | **10** | **20** | **40** | **10** | **20** | **100** | **Tb**  (-) | **Wb** (-3)^c^ | **R/C**  (-) | **Iz** (-3)^c^ |
| Bs2WT | + | + /- | + / + | + / + | + / - | - /- | - /- | - /- | - | 0 | - | 0 |
| Bs2Δ*wadB* | + | + /- | + / + | + / + | + / - | - /- | - /- | - /- | - | 0 | - | 0 |
| Bs2Δ*wadD* | + | + /- | + / + | + / + | + / - | - /- | - /- | - /- | - | 0 | - | 0 |
| Bs2Δ*ppdK* | + | + /- | + / + | + / + | + / - | - /- | - /- | - /- | - | 0 | - | 0 |
| Bs2Δ*ppdK*Δ*wadB* | + | + /- | + / + | + / + | + / - | - /- | - /- | - /- | - | -1 | - | 0 |
| Bs2Δ*ppdK*Δ*wadD* | + | + /- | + / + | + / + | + / - | - /- | - /- | - /- | - | -1 | - | 0 |
| Bs2Δ*wbkF* | + | + /- | + / + | + / + | + / - | - /- | - /- | - /- | - | - | - | - |
| Bs2Δ*wzm* | + | + /- | + / + | + / + | + / - | - /- | - /- | - /- | - | - | - | - |
| Bs2::Tn7Km^R^ | + | + /- | + / + | + / + | + / - | - /- | - /- | - /- | - | 0 | - | 0 |

1. Phages: Tbilisi (Tb), Weybridge (Wb), R/C and Izatnagar (Iz)
2. RTD: routine test dilution (maximum dilution of the phage to which the *B. suis* 1330 reference strain is lysed
3. The numbers correspond to the maximum dilution of the phage to which the strains are lysed
