# Supplementary material for: Development of attenuated live vaccine candidates against swine brucellosis in a non-zoonotic B. suis biovar 2 background
Source: Vet Res. 2020 Jul 23;51:92. doi: 10.1186/s13567-020-00815-8 (PMC7376850; doi:10.1186/s13567-020-00815-8)
Supplement: Supplementary file 6 — Additional file 6. Bs2WTpckAframeshift is in a position different from that in otherBrucellaspecies that carry a mutated PckA. Alignment of PckA of B. microti and B. suis biovar 5, B. melitensis 16M and B. abortus 2308, B. suis bv2 Thomsen and Bs2WT. The amino acids differing are indicated in bold and shaded in gray. [file 13567_2020_815_MOESM6_ESM.pptx]

## Slide 1
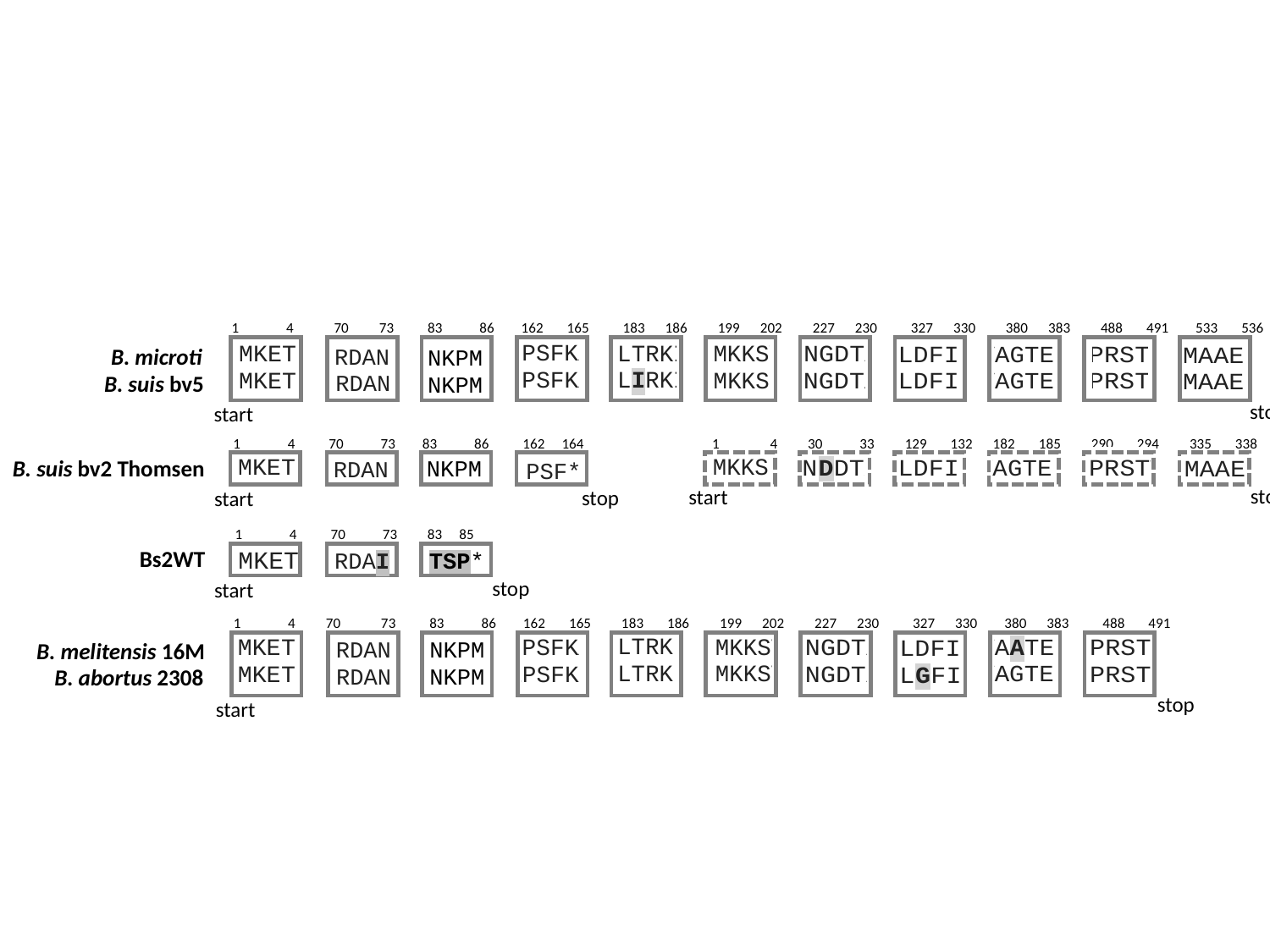

1 4 70 73 83 86 162 165 183 186 199 202 227 230 327 330 380 383 488 491 533 536
RDAN
RDAN
B. microti
NKPM
NKPM
B. suis bv5
stop
start
1 4 70 73 83 86 162 164 1 4 30 33 129 132 182 185 290 294 335 338
PSF*
B. suis bv2 Thomsen
NKPM
RDAN
start
stop
start
stop
1 4 70 73 83 85
Bs2WT
TSP*
RDAI
stop
start
1 4 70 73 83 86 162 165 183 186 199 202 227 230 327 330 380 383 488 491
NKPM
NKPM
RDAN
RDAN
B. melitensis 16M
B. abortus 2308
stop
start
